# Pericardial involvement in neoplastic diseases

**DOI:** 10.1590/1806-9282.2024S105

**Published:** 2024-06-07

**Authors:** Henrique Murad, João Carlos Ferreira Leal, Rui Manuel de Sousa Sequeira Antunes de Almeida, Vinicius José da Silva Nina

**Affiliations:** 1 Universidade Federal do Rio de Janeiro, Brazilian Society of Cardiovascular Surgery – Rio de Janeiro (RJ), Brazil.; 2 Universidade Estadual de São Paulo, São José do Rio Preto Medical School, Brazilian Society of Cardiovascular Surgery – São José do Rio Preto (SP), Brazil.; 3 Medical School of the Assis Gurgacz Foundation University Center, Brazilian Society of Cardiovascular Surgery – Cascavel (PR), Brazil.; 4 Universidade Federal do Maranhão, Brazilian Society of Cardiovascular Surgery – São Luis (MA), Brazil.

## INTRODUCTION

There are several ways of pericardial involvement in neoplastic diseases: acute pericarditis, pericardial effusion, cardiac tamponade, and constrictive pericarditis^1^.

Primary tumors of the pericardium, such as mesotheliomas, sarcomas, or teratomas, are very rare. Secondary involvement of the pericardium by primary tumors elsewhere is much more common, mainly from lungs, breasts, blood, and mediastinal lymph nodes. Pleural mesotheliomas and gastrointestinal cancers may also evolve with pericardial effusion^2,3^. Pericardial neoplastic involvement is a predictor of poor prognosis, and most treatments are palliative^4^. Pericardial effusion may be the first marker of occult cancer and is correlated with advanced stages of the disease^5^. A study by Fernandes et al., revealed that 16.9% of 254 cases of pericardial effusion had a neoplastic etiology^6^. Patients with neoplastic pericardial effusion typically have a life expectancy of less than 4 months^7^.

Patients with neoplasms can develop pericardial disease due to non-neoplastic etiologies, such as those induced by radiotherapy, chemotherapy, infections in immunocompromised patients, and autoimmune and idiopathic causes.

## PERICARDITIS

Acute pericarditis is an inflammatory process that may present effusion and has the following clinical features: (1) acute retrosternal chest pain exacerbated by breathing and relieved when sitting up; (2) a pericardial rub audible at the left sternal border; (3) diffuse ST-segment elevation and PR-segment depression on the electrocardiogram (EKG); and (4) pericardial effusion.

This condition has a self-limited evolution and is treated with acetylsalicylic acid and non-steroidal anti-inflammatory drugs. Low-dose colchicine is advised to enhance the effectiveness of the prescribed medications and to avoid recurrence. A major investigation is needed in cases involving high fever, subacute evolution, major pericardial effusion, cardiac tamponade, and no response to medical treatment^8^.

## PERICARDIAL EFFUSION

The majority of patients with pericardial effusion, even those with large effusions, are asymptomatic, as the effusion evolves slowly and insidiously. Diagnosis is commonly made by routine chest X-rays and echocardiogram.

Common symptoms, when present, are cough, dyspnea, and pleuritic pain. In rare cases, patients may present palpitation due to low cardiac output, dysphagia due to esophagus compression, hoarseness due to recurrent laryngeal nerve compression, and hiccups due to phrenic nerve compression^3,8^.

A chest X-ray may show cardiomegaly with a flask-shaped heart and clear lungs without pleural effusion or lung congestion. EKG can be normal or present non-specific ST segment and T-wave elevation.

**Figure 1:**
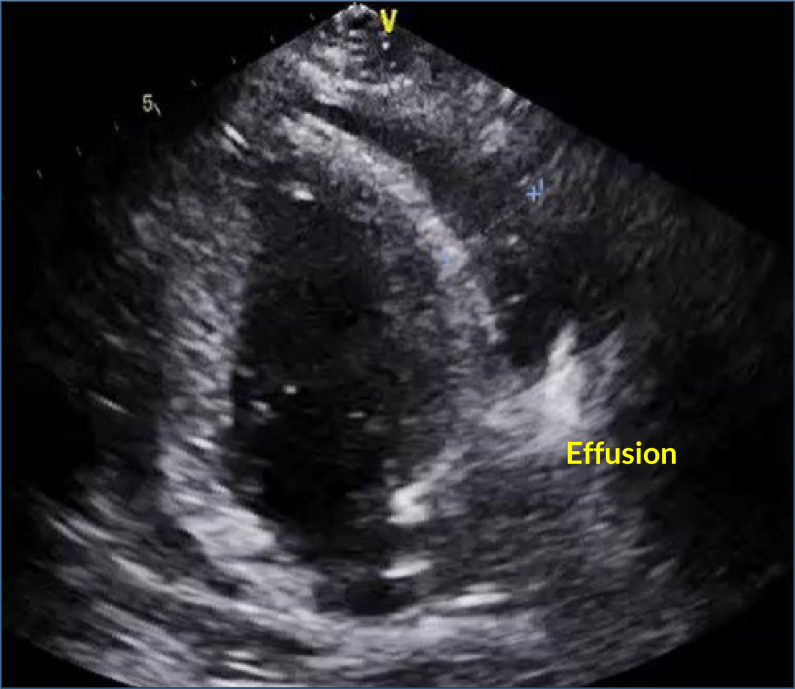
Pericardial effusion in a breast cancer patient (image courtesy of Dr. Márcio Mendes).

**Figure 2:**
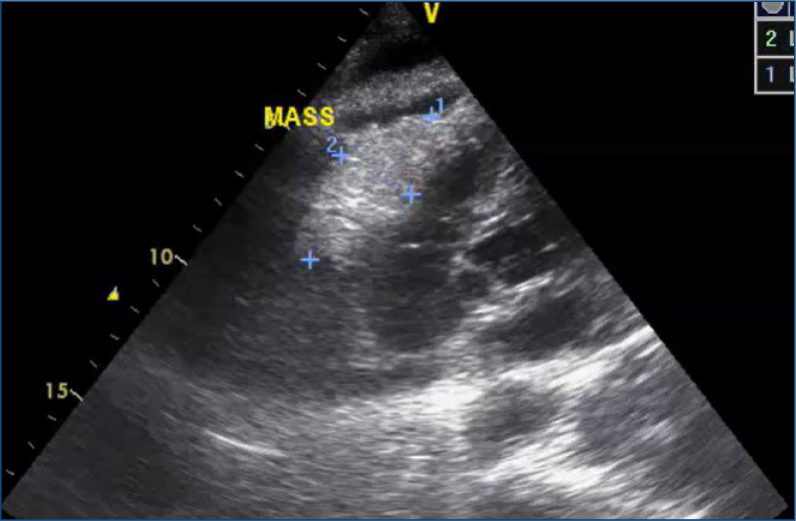
Pericardial metastasis from gastric cancer (image courtesy of Dr. Márcio Mendes).

Echocardiogram is the most important diagnostic test for detecting pericardial effusion (Figure 1). It assesses effusion volume, location, hemodynamic impairment, and eventual cardiac tamponade. An echocardiogram can also detect neoplastic intrapericardial masses (Figure 2).

Computed tomography and magnetic resonance imaging can be used to detect and evaluate primary tumors such as lung cancer, loculated effusion, pericardial thickness, and malignant tumor deposits in the pericardial sac^1,8,9^.

## CARDIAC TAMPONADE

A large pericardial effusion can lead to cardiac tamponade, which is a condition where the pericardial liquid pressure exceeds the ventricular filling pressure, resulting in low cardiac output and cardiogenic shock. As the neoplastic pericardial effusion has a subacute slow filling of the pericardial sac, only few patients present the classic Beck's triad of arterial hypotension, tachycardia, and muffled heart sounds.

Patients with cardiac tamponade commonly present neck vein distension with a rise in jugular pressure that does not decrease during deep inspiration (known as Kussmaul's sign). There is also a drop of more than 10 mmHg in systolic arterial pressure during deep inspiration (*pulsus paradoxus*)^1,9^.

On an EKG, diffuse low voltage may be observed.

Echocardiography is crucial for diagnosing and evaluating the severity of pericardial tamponade. Several key echocardiographic findings in pericardial tamponade include (a) late right atrium systolic collapse; (b) early right ventricle diastolic collapse; (c) interventricular septal bulge to the left during inspiration and reversal movement during expiration; and (d) inferior vena cava dilation without inspiratory collapse.

## CONSTRICTIVE PERICARDITIS

Constrictive pericarditis can arise as a complication of radiotherapy for breast cancer, leukemia, or lymphoma. Patients usually present with fatigue, dyspnea, palpitations, and ascites. Computed tomography and cardiac magnetic resonance are very useful in the diagnosis and evaluation of constrictive pericarditis^10^.

## SURGICAL TREATMENT

The main objectives of the surgical treatment for neoplastic pericardial involvement with effusion or constriction are symptom relief and hemodynamic stability. Those objectives can be obtained through pericardiocentesis, pericardial window, and pericardiectomy. Adjunctive therapy is used mainly to avoid recurrence^11^.

## PERICARDIOCENTESIS

Pericardiocentesis is the treatment of choice for cardiac tamponade and large effusions. Besides fluid removal, another benefit of pericardiocentesis is the cytological analysis of pericardial fluid to detect neoplastic cells.

The procedure should be guided by echocardiography or fluoroscopy. A needle is advanced either subxiphoidally or near the area of major pericardial fluid collection close to the chest wall until fluid is aspirated. After the initial syringe fluid aspiration, a multiperforated pigtail catheter is introduced into the pericardial sac by the Seldinger technique to drain the remaining pericardial effusion.

The pigtail catheter is kept in the pericardial sac under low-pressure aspiration until the drainage amount is <30 mL/day, with the objective of reducing recurrence. Neoplastic pericardial effusion can have a recurrence rate of up to 70%^12^.

## PERICARDIAL WINDOW

The subxiphoid pericardial window is a commonly used procedure that allows pericardial fluid drainage and pericardial biopsy. This window is created through an epigastric incision under local or general anesthesia. The pericardium can be approached in a space developed below the distal third of the sternum.

A pleuropericardial window enables drainage of pericardial tamponade and large effusions into the pleural space and pericardial biopsy. This window can be done through a small thoracotomy or preferably through video-assisted thoracoscopy. While these procedures provide palliative relief, pericardial effusion can recur, mainly due to fluid loculation^9^.

A pleuropericardial window can also be achieved through percutaneous thoracic puncture and posterior balloon enlargement of the pericardial puncture site^13^.

## PERICARDIECTOMY

Pericardiectomy, whether partial or total, might be considered in cases of constrictive pericarditis or recurrent pericardial effusion that persists after pericardiocentesis and pericardial window. This procedure is rarely performed and is associated with a high mortality rate. The decision for pericardiectomy depends on the type of tumor, tumor stage, and surgical risk to the patient^10,14^.

## ADJUVANT THERAPY

Every patient with neoplastic pericardial effusion should be treated with systemic antineoplastic agents. Cytotoxic agents should be instilled into the pericardial sac to avoid recurrence. Celik et al., found that patients who underwent a pericardial window procedure plus chemotherapy fared better than those receiving pericardiocentesis plus chemotherapy^15^. For primary lung cancer, cisplatin is the preferred choice, while thiotepa is used for breast cancer. Intrapericardial tetracycline can be used as a sclerosing agent to avoid recurrence, but it often leads to arrhythmias, chest pain, and high fever^16^.

Radiotherapy can be used in cases of lymphomas and leukemia^8^.

## KEY MESSAGES

1. Secondary pericardial involvement is common in neoplastic diseases.
2. Pericardial effusion in neoplastic patients may have other etiologies beyond cancer itself.
3. Pericardial primary tumors are rare. Pericardial secondary tumors most frequently originate from lungs, breasts, mediastinal lymphomas, and leukemias.
4. Pericardial involvement in neoplastic diseases can cause pericarditis, pericardial effusion, cardiac tamponade, and rarely constrictive pericarditis.
5. Echocardiogram is the most important test for diagnosing neoplastic pericardial disease. A definitive diagnosis is made by cytology or pericardial biopsy. Investigation of the primary tumor is mandatory.
6. The basis of the treatment includes systemic antineoplastic drugs, pericardiocentesis for diagnosis and symptom relief, and intrapericardial instillation of antineoplastic agents.
7. Pericardial effusion is treated by pericardiocentesis and pericardial window but has a high recurrence rate. In some cases, pericardiectomy may be needed.
